# Vertically- and horizontally-transmitted memories – the fading boundaries between regeneration and inheritance in planaria

**DOI:** 10.1242/bio.020149

**Published:** 2016-08-26

**Authors:** Moran Neuhof, Michael Levin, Oded Rechavi

**Affiliations:** 1 Department of Neurobiology, Wise Faculty of Life Sciences, Tel Aviv University, Tel Aviv 69978, Israel; 2 Allen Discovery Center, Tufts University, 200 Boston Avenue, Suite 4600, Medford, MA 02155, USA; 3 Sagol School of Neuroscience, Tel Aviv University, Tel Aviv 69978, Israel

**Keywords:** Planaria, Regeneration, Memory, Inheritance, Epigenetics, Evolution, Generations, Transgenerational, Small RNAs, Chromatin

## Abstract

The Weismann barrier postulates that genetic information passes only from the germline to the soma and not in reverse, thus providing an obstacle to the inheritance of acquired traits. Certain organisms such as planaria – flatworms that can reproduce through asymmetric fission – avoid the limitations of this barrier, thus blurring the distinction between the processes of inheritance and development. In this paper, we re-evaluate canonical ideas about the interaction between developmental, genetic and evolutionary processes through the lens of planaria. Biased distribution of epigenetic effects in asymmetrically produced parts of a regenerating organism could increase variation and therefore affect the species' evolution. The maintenance and fixing of somatic experiences, encoded via stable biochemical or physiological states, may contribute to evolutionary processes in the absence of classically defined generations. We discuss different mechanisms that could induce asymmetry between the two organisms that eventually develop from the regenerating parts, including one particularly fascinating source – the potential capacity of the brain to produce long-lasting epigenetic changes.

## Abstract (precis)

**Summary:** In this hypothesis paper we re-evaluate canonical ideas about the interaction between developmental, genetic and evolutionary processes through the lens of planaria, an invertebrate model organism which challenges fundamental assumptions regarding reproduction.

## Introduction

Most models of evolution, which are based on Mendelian genetics, depend conceptually on the existence of a distinct separation between generations across an ancestry. This distinction between parents and children is supposedly enforced by Weismann's barrier, which in theory precludes information transfer from the soma to the germline, and thus prevents inheritance of parentally-acquired traits (Poulton et al., 1889; Sabour and Schöler, 2012). The germline, according to this framework, is conceived as a ‘bottleneck’, which filters out epigenetic responses. In other words, all the changes that affect somatic cells, whether epigenetic or genetic (e.g. mutations, transpositions), are erased in the next generation. Lamarck's discarded theory of evolution, according to which somatic responses (and acquired traits) are carried over to the progeny, assumed a continuation between the generations, and until recently was considered to be entirely incorrect (Jablonka and Lamb, 2015). New discoveries in the field of epigenetics, some of which will be discussed here, suggest the need for reexamination of these original ideas in a new light.

As unicellular organisms have been shown to preserve cellular states over generations (Zacharioudakis et al., 2007), Weismann's barrier as originally suggested is relevant to organisms that have a well-defined and segregated germline (namely, only specific, designated cells will become germ cells). However, do similar restrictions on the process of evolution apply to plants (where the germline is not segregated), or to the many phyla of animals that can reproduce asexually without going through a germline bottleneck?

Even in metazoans, which segregate their germline and for which Weismann's barrier is supposedly relevant, different mechanisms are used to specify the primordial germ cells (Extavour, 2003). These different mechanisms allow different degrees of communication between the parent's environment and the germline. Recent evidence suggests that the variance between germline specification mechanisms could influence the process of evolution, and specifically, that a continuity with the previous generation could accelerate evolution. For example, it was shown that genes evolve faster in amphibians that define their germline by using maternally inherited determinants (‘preformation’), in comparison to the rates of gene evolution seen in related organisms that define their germline by inductive signals (‘epigenesis’), without inheriting ‘germplasm’ (which should be affected by the environment) from the mother (Evans et al., 2014).

One asexually reproducing animal, on which we will focus in this paper, which presents an interesting challenge to the Weismann barrier, is planaria. Planarians are an order of free-living flatworms which are complex bilaterians possessing a wide range of cell types, a true centralized brain, and a complex repertoire of behavioral responses (Saló et al., 2009). Planaria have advanced mechanisms of regeneration, and are able to coordinate their resident population of stem cells to recreate any portion of the animal that is surgically removed, including their brain, throughout adulthood (Roberts-Galbraith and Newmark, 2015). These attributes have made it a popular model system for studies of stem cell regulation, morphogenesis, behavioral plasticity, and physiological signaling (Gentile et al., 2011; Nicolas et al., 2008; Shomrat and Levin, 2013).

While many of the common planarian species (which are grown in the lab and are considered model organisms for regeneration) can reproduce sexually (Cardona et al., 2006), they most frequently reproduce asexually through fission followed by regeneration. Upon bisection (whether externally induced or self-initiated), a structure called the ‘blastema’ forms in each fragment (Birnbaum and Sánchez Alvarado, 2008). The blastema gives rise to new tissues, and a process of remodeling then scales both new and existing structures appropriately (Beane et al., 2013). When a head fragment regenerates its missing tail, or when a tail fragment regenerates a missing head, new cells differentiate from pluripotent stem cells known as ‘neoblasts’. These unique cells are required for regeneration, and also for the continuous remodeling and morphological rescaling observed in intact worms during growth and starvation (Oviedo et al., 2003). The neoblasts are instructed both by intrinsic state (cell-autonomous pathways) and information from surrounding cells (Oviedo and Levin, 2007; Oviedo et al., 2010; Wagner et al., 2011; Witchley et al., 2013).

Here, we explore a number of scenarios that could potentially defy classical models of evolution. Specifically, we ask whether in planaria and other organisms that reproduce by fission, different types of epigenetic information are asymmetrically passed across generations. Such stored information, which can be regarded as memory (see more below and in the glossary), could play many crucial roles in regulating behavioral and developmental patterns. In this manuscript we will discuss different types of memories that may persist upon regeneration/inheritance; memories of gene activity, memories which are encoded in the connectivity of neuronal circuits, and memories of non-neural physiological states.

In the broadest sense of the word, memory is what enables altering of future responses based on history. Biological memory is encoded at many levels: metabolic differences (Cameron et al., 2012; Ros et al., 2006), epigenetic factors (e.g. small RNAs, histone marks, DNA methylation and prions) (Bird, 2002; D'Urso and Brickner, 2014; Iwasaki and Paszkowski, 2014), stable bioelectrical circuit modes (Cervera et al., 2014; Law and Levin, 2015), or neuronally-encoded memories (Axmacher et al., 2006; Daoudal and Debanne, 2003; Herry and Johansen, 2014; Maren and Quirk, 2004; Zhang and Linden, 2003). A myriad of mechanisms exist to allow molecules, molecular pathways, cells, and cellular networks to transduce physiological or behavioral inputs (experiences) into stable state changes that guide future activities. In this sense, processes that ensure the persistence of different developmental fates or trajectories are also forms of memory.

The Weismann barrier is relevant to asexual organisms as well, because the issue is not only which cells will contribute to the next generation, but whether and how the life history of the body gets permanently encoded in cells so as to significantly alter the offspring in a stable manner. Indeed, the potential breaching of the Weismann barrier in planaria has previously been considered, in the context of tracking the source of the cellular contents of neoblasts that form a new organism (Solana, 2013). However, could parentally-produced alterations that encode biological memory breach Weismann's barrier and persist across generations? Even if information could travel from somatic tissues to the germline, several rounds of reprogramming events (in the germline and in the embryo) were previously thought to prevent the inheritance of epigenetic memory in animals (Mann and Bartolomei, 2002; Messerschmidt et al., 2014; Morgan et al., 2005; Vucetic et al., 2010). Nevertheless, in recent years it has become clear that complex and still poorly understood regulatory processes determine which epigenetic memories would persist, and which would be erased across generations. The removal of DNA cytosine methylation and histone marks during embryogenesis was thought to ‘clean’ the embryo of epigenetic modifications that were present on its parents' genome. The addition of *de novo* chromatin modifications in the next generation was similarly thought to depend solely on the current environmental conditions, and the dictation of the hard-wired, genomically-encoded developmental program. However, reprogramming is not complete and a few parental marks escape removal (Hackett et al., 2013).

How widespread are heritable memories and what types of memories avoid reprogramming? We will explore these questions through planaria, by focusing on the events that take place when animals reproduce by fission.

Glossary **Memory**: retention of information about a state of affairs for some time period; the ability of a system to specifically alter some aspect of a labile medium in response to stimuli, such that future responses to stimuli are altered. Memory requires latency between stimulus and salient response.

**Epigenetic modifications**: defined here as factors that alter the phenotype that are not stored in the genetic code, including but not limited to DNA methylation, histone modifications and small RNAs.

**Bioelectric network/circuit**: a group of cells, not restricted to neurons/muscle, often connected by gap junctions, which communicate via slow changes in resting potential and endogenous electric fields, which regulates cell state and large-scale morphogenesis.

**Maternal effects**: factors that alter the phenotype of the progeny that depend on the maternal environment, including genetic, epigenetic and physiological effects.

**Epimutations**: as opposed to DNA mutation, an epimutation is a molecular alteration to the DNA that does not alter the DNA sequence that can be stably transmitted across generations. Most commonly refers to differences in cytosine methylations between certain alleles. Epimutations can be segregated with the chromosomes in accordance with Mendel's roles.

**Plant embryo**: a phylogenetically conserved structure that develops from the zygote containing the shoot and root apical meristems, and the primordial tissues that will differentiate into tissues of the mature plant.

**Meristem**: in plant biology, meristems are self-maintaining structures of undifferentiated cells from which plant organs develop.

## Hypothesis

We hypothesize that the asymmetric fission of planaria, and similar organisms, and the resulting genetic and epigenetic differences in the individuals that regenerate from the different fragments, can create stable variation and therefore participate in the process of evolution.

## Reproduction as regeneration

A generation can be defined as ‘a single step in natural descent’ (http://www.dictionary.com/, accessed 2015). In planarian asexual reproduction, this definition does not necessarily apply, since after fission the relationship between the two resulting individuals does not display a clear hierarchy – which half is the ‘parent’ and which half is the ‘child’? Is one half ‘older’ than the other? Despite these ambiguities, we suggest that parentally-acquired information (the result of the parent's life experiences) could be transmitted from the worm that underwent splitting to the two organisms that form upon regeneration, and therefore the term ‘inheritance’ is relevant when discussing fission. The term ‘genetics’ could also be relevant in this regard, although, as will be elaborated below, the information that is inherited from the parent might not be restricted to changes in genes.

Fission and regeneration in planaria involve long-range instructive communication among cells (a signaling mode that can facilitate breaches of Weismann's barrier). When a worm is bisected, cells on the anterior- and posterior-facing sides of the cut must form a tail and head, respectively; the cut plane separates cells that were adjacent neighbors, and therefore had essentially the same positional information, yet these generate completely different anatomical structures. Thus, cell position (the local microenvironment) does not uniquely dictate the appropriate morphological outcome; instead, cells must communicate with other remaining tissues in order to determine which structures each blastema needs to build (Nogi and Levin, 2005; Oviedo et al., 2010; Reddien and Sánchez Alvarado, 2004). A similar long-range, highly integrated pattern control is seen in amphibians, where tails transplanted to the side of a salamander eventually remodel to limbs (including the transformation of the tail tip into fingers, which reveals that tissues can change their morphological structure in response to global patterning cues) (Farinella-Ferruzza, 1956).

The process of regeneration is essentially one of cell networks processing information about large-scale growth and form. A focus on information reveals an interesting analogy between generational descent and regeneration; that of *space* versus *time*. With classical generational inheritance, patterning information is passed on *temporally* from parent to offspring via the genome, conserved with high fidelity and yet susceptible to environmental influence. In regeneration, in addition to its temporal progression, instructive information is also propagated *spatially*, from the rest of the body to a wound region and thus to new tissues; planarian regeneration is a remarkable example of how these two distinct but highly parallel pattern control processes converge. It should be noted that while we focus on planaria as a uniquely tractable model for these studies, stable modifications to regenerative pattern occur also in mammals (trophic memory in deer antlers) and other invertebrate systems such as crab limbs (reviewed in Lobo et al., 2014).

The parallelism between development and regeneration is also seen at the cellular level, as manifested in the similarities between germ cells and the stem cells that enable regeneration in planaria (Solana, 2013). In asexual reproduction, both tail and head fragments regenerate their missing tissues through the proliferation and differentiation of neoblasts. Thus, when planarians reproduce asexually, the new generation does not originate from one cell, but from a ‘community of cells’ (generation/regeneration of a worm from a single neoblast without a surrounding mature body has never been shown). Because genomic changes arise during cell division, and as a result of DNA damage of different sorts, this ‘cell community’ is expected to be composed of a mixture of different neoblasts, and also from genomically-different surrounding cells, which are not totipotent. It was recently demonstrated that the different neoblasts are not completely genetically identical – even in the same individual, a large number of mutations and SNPs differentiate between neoblasts (Nishimura et al., 2015). Moreover, it is not clear that the information that is required for regeneration (where, when, and how much to make of the new cell types, how to arrange those new tissues in correct geometric patterns, and crucially, when to stop growing) is present in the neoblasts; thus, the genetic variance in the surrounding cells could also be crucial, and differentiate the organisms that grow from the two regenerating halves. The barriers to the interaction between the surrounding cells and the neoblasts are also analogical to the Weismann barrier, between somatic cells and germ cells.

Similarly to the germ cells of other animals, planarian neoblasts (unlike other cell types) express PIWI homologues (Friedländer et al., 2009). In other organisms, PIWI proteins, and PIWI-associated small RNAs, or piRNAs, are important for maintaining the immortality of the germline (Meister, 2013), and their role in somatic tissues in less clear (Rajasethupathy et al., 2012). In *Caenorhabditis* *elegans*, for example, animals without a germline are virtually devoid of piRNAs (Bagijn et al., 2012). PIWI proteins and piRNAs play a critical role in the silencing of transposons and enable distinction between ‘self’ and ‘foreign’ genes, and therefore preserve the progeny's genome (Rechavi, 2014). The heritable small RNA pool, which includes piRNAs and other types of small RNAs (e.g. endo-siRNAs in *C. elegans*) (Claycomb, 2014; Gent et al., 2010; Rechavi et al., 2014; Vasale et al., 2010), and tRNA-fragments in mice (Chen et al., 2016; Liao et al., 2014; Peng et al., 2012; Sharma and Rando, 2014), constitutes a germline ‘memory bank’ of sequences that were found in past generations to be ‘dangerous’ (mobile parasitic DNA elements) or ‘safe’ (genes that need to be expressed in the germline). Transmission of piRNAs to progeny ensures that transposons will not jump, thus preventing disruption of the germline's genome, and ensuring error-proof transgenerational information transfer (Malone and Hannon, 2009). Neoblasts, which grant planarians their powerful ability to regenerate endlessly, express PIWI proteins and piRNAs (Reddien et al., 2005), and were recently shown, like germ cells, to use piRNAs to preserve the integrity of their genomic heritage (Zhou et al., 2015).

## Asymmetry and memory

Asymmetric retention or erasure of cellular memory, after cell division, is an important and well-studied mechanism in development, crucial both for renewal of pluripotency/proliferation, and for differentiation and establishment of cell fate (Armakolas et al., 2010; Di Laurenzio et al., 1996; Jan and Jan, 1998; Klar, 1987). Asymmetric cell division (in neurons and other cell types) is also used as a mechanism for preventing aggregated, damaged or misfolded proteins from being inherited to the cell progeny by confining them to only one daughter-cell (Ogrodnik et al., 2014). A similar phenomenon is familiar in budding yeast, where asymmetric division results in two daughter-cells; one of them contains large amounts of unfolded and aggregated proteins, usually associated with aging, while the other remains ‘young’ (Spokoini et al., 2012).

Similarly, asymmetric fission of an entire multicellular organism, such as planaria, could result in asymmetric inheritance of cells which, in theory, could have distinct expression patterns maintained by cell-specific epigenetic states. Could the uneven inheritance of epigenetic effects make the organisms that develop from the two separate fragments phenotypically unequal?

Indeed, planarian ‘clones’ that regenerate from fragments of a single animal and that live in the same container, can show variable responses to an external perturbation such as a pharmacological compound (Beane et al., 2011; Chan et al., 2014; Oviedo et al., 2010). At the molecular level, fission and the ensuing recreation of a new individual in planarians may not necessarily entail complete ‘resetting’ of modifications (such as histone marks, RNA content and synaptic connection strengths) that were acquired by the previous ‘generation’. Asymmetric fission could therefore be a mechanism that enables retention of life history memories; some epigenetic changes, specific to the tail or head sections, may persist, at least in the tissues that were not regenerated anew. As a result of these retained memories of the ancestor's gene activity, the resulting individuals might respond differentially to changes in the environment in the future. If indeed epigenetic marks are asymmetrically distributed, whether through a passive/random process, or via active mechanisms (similarly to the mechanisms that asymmetrically distribute aggregated proteins in dividing neurons or yeast, that were described above), then we suggest that the clonality of the resulting individuals should be questioned, and that the evolution of the species could be affected.

## Which memories might survive fission?

Therefore, are all clones created equal, or could epigenetic information survive splitting? The answer depends on the capacity of asymmetric fission to maintain long-term variability – the ability of each cloning product (each ‘individual’) to hold memories acquired by their ancestral body (or the relevant part thereof) in its lifetime. A few different mechanisms, which are not mutually exclusive, and could operate in tandem, could in theory establish asymmetry following planarian fission.

### Genetic diversity in the progenitor cell population

Since the new individual is regenerated from a ‘community of cells’ and not from one unique cell, asymmetric fission could non-randomly distribute genetically distinct neoblasts to the two fragments. The asymmetry in this regard may not be entirely random; genetic variability could be caused by differential mutation rates in different tissues of the body; it was suggested that neurons, for example, display more genetic variability (Muotri and Gage, 2006).

In theory, since the different genomes are packed into different cells, which do not fuse, the genetics of planaria that reproduce by fission could be dictated by the frequencies of multiple non-recombining alleles that are present within a single organism. This possible mosaicism also has practical considerations for planaria geneticists. Since each worm is created from multiple ‘germline-like’ neoblasts, genetic editing of an entire worm's genome (by CRISPR for instance) would require manipulation of *all* the neoblasts' genomes, or highly efficient selection of those neoblasts which were successfully edited; otherwise, only a mosaic animal would be achieved. Indeed, a recent study reveals that genetic mosaicism in planarian cells can create genetic diversity in a population of asexually reproducing animals (Nishimura et al., 2015).

### Biochemical gradients

Following splitting, each fragment obtains a different composition of molecules (e.g. proteins, RNA molecules, gradients of morphogens) (Adell et al., 2010), which influence and guide its subsequent physiology and regeneration. The existence of such gradients and local environments in the worm may contribute to the initial state of the newly-formed fragments. In addition to short-term immediately derived ‘maternal effects’, long-term effects, amplified by positive feedback processes, could perpetuate after ‘maternal’ factors are diluted. It must be noted that such gradients have to be self-scaling, to maintain their instructive pattern within the resulting small fragments (Ben-Zvi and Shilo, 2011; Werner et al., 2015).

### Epigenetic mechanisms in planaria

Epigenetic mechanisms in planarian neoblasts are currently being explored (Duncan et al., 2015; Hubert et al., 2013; Robb and Sánchez Alvarado, 2014; Rouhana et al., 2014). If different environmental events affect small RNA pools (microRNAs and piRNAs have been described in planaria) or chromatin modifications in a spatially restricted manner, then such epigenetic processes, which in a number of organisms perpetuate transgenerational gene regulation, could mediate asymmetry following fission. Interestingly, as is the case in *C. elegans* nematodes and in plants, RNA interference (RNAi) works systemically in planaria (Rouhana et al., 2013). Thus, in theory, small RNAs could allow both spatial and temporal spreading of epigenetic memory in planaria.

### Somatic effects on neoblasts

As neoblasts are influenced by information received from other somatic cells around them (Oviedo and Levin, 2007), the practical meaning is that in planaria a breaching of Weismann's barrier could take place. While neoblasts are thought to drive regeneration, the anatomical outcomes they implement are regulated by gap junctional coupling and neural inputs from other cells (Oviedo et al., 2010); however, it is unclear precisely which elements of patterning information are intrinsic to the stem cell and which are computed by interactions with surrounding cells and the environment. If the neoblasts are indeed influenced by somatic cells while regenerating, somatic cells may be involved in determining the phenotype of the new individual.

Communication of somatic cells with neoblasts could be mediated by multiple mediators (e.g. hormones, small RNAs, ionic signaling). One common solution for coordinating the activity of cell networks is the use of gap junctions (electrical synapses that underlie plasticity in networks, both neural and non-neural) (Palacios-Prado and Bukauskas, 2009; Pereda et al., 2013). Such channels were directly shown to be required for neoblast function (Oviedo and Levin, 2007). Gap junctions are critical for cell-cell communication in embryogenesis (reviewed in Mathews and Levin, 2016) and in patterning disruptions such as cancer (Mesnil et al., 2005; Trosko, 2007; Yamasaki et al., 1999); this is well-conserved, from invertebrates through man, including the regulation of stem cell activity by gap junction-dependent signals (Jäderstad et al., 2010; Todorova et al., 2008; Wolvetang et al., 2007; Wong et al., 2008). Because they determine a cell's resting potential (by allowing electrical inputs from neighboring cells) but are themselves voltage-gated, they implement positive feedback loops that are an ideal mechanism for stabilizing physiological signals as stable memories (Levin, 2014b; Palacios-Prado and Bukauskas, 2009). It is thus no accident that brains capitalize extensively on gap junction-mediated plasticity for learning and memory in the CNS (Allen et al., 2011; Maciunas et al., 2016; Wang and Belousov, 2011; Wu et al., 2011).

### Bioelectric circuits and somatic pattern memory

Recent work has begun to reveal that patterns of resting potential differences across cell groups *in vivo* specify aspects of large-scale pattern formation during development and regeneration (reviewed in Levin, 2012, 2014b). Memory in the CNS is thought to involve synaptic plasticity implemented by neurotransmitters, ion channels and gap junctions (electric synapses) (Bailey and Kandel, 2008; He et al., 2014; Pereda et al., 2013). However not only neurons and muscle cells possess these proteins and the ability to communicate electrically (Bates, 2015; Funk, 2013; Sundelacruz et al., 2009). Slow changes in resting potential (not millisecond-rate spiking) regulate proliferation, differentiation, apoptosis and migration in a range of somatic and stem cells (reviewed in Blackiston et al., 2009; Funk, 2015; Sundelacruz et al., 2009). The dynamics of these bioelectric circuits implement signals that trigger or suppress regeneration (Adams et al., 2007; Jenkins et al., 1996; Tseng et al., 2010). In both embryogenesis and regeneration, endogenous spatial gradients of these potentials across tissues and anatomical axes coordinate aspects of large-sale patterning, including stem cell differentiation (Sundelacruz et al., 2008, 2013), size control (Beane et al., 2013; Perathoner et al., 2014), polarity of the left-right (Levin et al., 2002), dorso-ventral (Stern, 1987), and anterior-posterior (Beane et al., 2011) axes, and induction of organs such as eyes (Pai et al., 2012), limbs (Altizer et al., 2001), and brains (Pai et al., 2015), in a range of species from planaria to mammals.

Thus, many tissues (not only the brain) can keep a record of physiological experience in stable modifications of bioelectric circuits that impinge on form and function of the animal. Indeed, physiological circuits consisting of ion channels and electrical synapses have now been shown to underlie long-term cardiac memory, where stable changes of heart beat rhythm to a different pattern can be induced by transient physiological effects (Chakravarthy and Ghosh, 1997; Zoghi, 2004), changes of pancreas response due to patterns of physiological stimuli in type II diabetes (Goel and Mehta, 2013), and bone, where osteogenesis is induced as a long-lasting effect of use-dependent potentiation (Spencer and Genever, 2003; Turner et al., 2002). Even single cells can stably store bioelectric state (induced changes in their resting potential) as intrinsic plasticity commonly studied in neurons (Cervera et al., 2014; Law and Levin, 2015; Levin, 2014a; Williams et al., 2002). However, far more complex memory can be implemented in networks of electrically-active cells by synaptic plasticity; experience-dependent changes in the electrical connectivity (topology) of a tissue and resulting reverberating loops. In many tissues (including the brain), this is in part mediated by gap junctions; electrical synapses that are themselves voltage-sensitive, allowing physiological history to shape future cell interactions (Palacios-Prado and Bukauskas, 2012; Pereda et al., 2013).

We recently tested the ability of gap junctional communication in somatic cell networks to implement somatic memory in planaria (reviewed in Durant et al., 2016) by transiently reducing gap junctional connectivity among cells. This can be accomplished by RNAi targeting 3 distinct Innexin proteins (Oviedo et al., 2010), which resulted in a bipolar two-headed planarian; posterior wounds of middle fragments grew heads instead of tails. The same result can be achieved by a transient (2-day) inhibition of gap junction communication using a blocker such as octanol (Nogi and Levin, 2005). The benefit of this approach is that unlike RNAi, which persists in tissues for long periods of time, octanol leaves planarian tissues within 24 h (as shown by HPLC) (Oviedo et al., 2010).

Remarkably, two-headed worms derived from a brief exposure to octanol immediately after cutting, continue to regenerate as two-headed in future rounds of amputation without the presence of octanol. This may be a result of gap junction connections being stably altered through conventional synaptic plasticity, or whether gap junction connectivity is restored to a normal state after the effect is canalized into another medium (e.g. chromatin modification), or both.

The ability of a transient physiological modulator to stably change the target morphology (the shape to which planarian fragments regenerate upon damage) suggests that at least some aspect of pattern memory is encoded in physiological networks and can be re-written by life events. Related phenotypes have also been produced by altering neurotransmitter pathways (Chan et al., 2014) and voltage-mediated circuits in planaria (Beane et al., 2011; Nogi et al., 2009; Zhang et al., 2011), consistent with a conserved role for bioelectric modules exploited for adaptive, plastic control of cell behavior in the body and organism behavior in the brain (Pezzulo and Levin, 2015).

### Neuronally-encoded memories

The planarian brain can form complex associations, such as learning and utilizing a set of context-specific behaviors (Best and Rubinstein, 1962; Halas et al., 1962; Thompson and McConnell, 1955; Umesono and Agata, 2009). Planaria have a true centralized brain (Nakazawa et al., 2003; Sarnat and Netsky, 1985), and brainless fragments exhibit no internally-motivated behavior or complex responses. Long-term memory in a number of vertebrate and invertebrate species has been shown to survive massive brain remodeling and regeneration (reviewed in Blackiston et al., 2015). Controversial experiments conducted with planaria in the 60s and 70s, but also modern experiments that were properly controlled and conducted using fully-automated training and tracking (Shomrat and Levin, 2013), suggest the possibility that some type of neuronally produced memories (an association between food and the haptic characteristic of the plate) can survive decapitation. Specifically, the data show that tail fragments of trained worms can retain information acquired during learning phases of the worm's life (Corning, 1966; McConnell et al., 1959; Shomrat and Levin, 2013). While the mechanisms by which information is encoded, stored, and imprinted on the newly regenerating brain remain to be understood, these data show modifications induced in adulthood can propagate via this animal's most frequent mode of reproduction.

These experiments raise the intriguing possibility that experience-dependent modifications are not limited to the head, but present throughout the animal. As the CNS is known to control not only behavior but also pattern regulation (Kiortsis and Moraitou, 1965; Mondia et al., 2011; Singer, 1952), including in planaria (Oviedo et al., 2010), fragments that inherit distinct portions of the nervous system could exhibit not only varied behavior but potentially different anatomical structure. In some species of planaria, transient changes of bioelectric connectivity, in the absence of mutation or introduction of foreign genes (i.e. despite a constant, normal genomic sequence), induce the formation of head morphology, brain shape and neoblast distribution typical to other extant species of planaria (Emmons-Bell et al., 2015). These data suggest that species-specific anatomical pattern upon regeneration is a function not only of the organism's genomic sequence but also of physiological events impinging on the body. These changes, unlike the induction of the two-head phenotype using gap junction inhibitors, are transient, again supporting the hypothesis that the effects are mediated by epigenetic mechanisms.

## Asymmetric retention of neuronally encoded memory

**Fig. 1.**
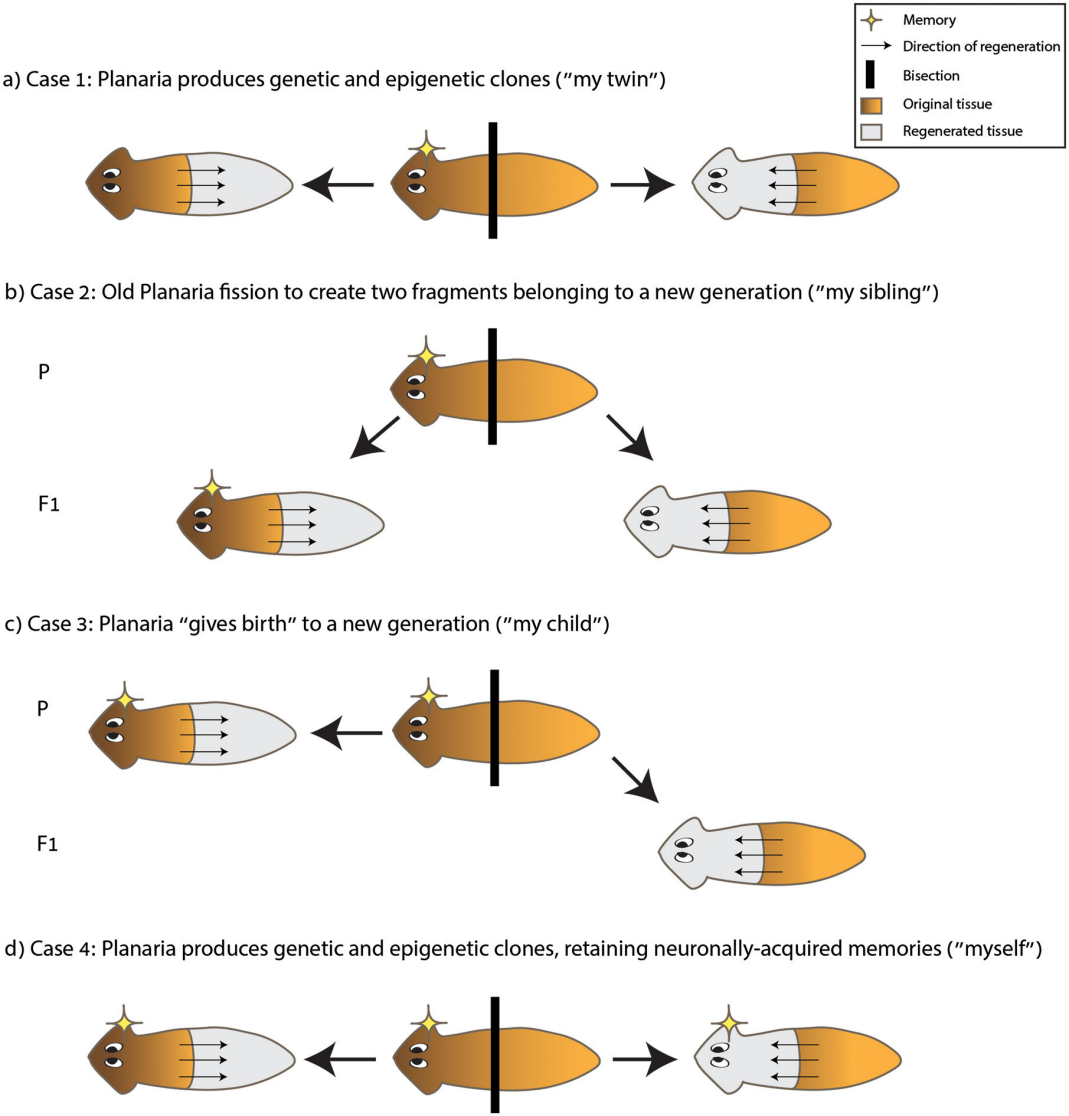
**Are all clones created equal?** Arrows mark the direction of regeneration, shades mark the regenerated part, and stars mark the retention of memory. (A) Case 1: planaria produces genetic and epigenetic clones upon fission, while brain-encoded memory is erased (‘twins’). (B) Case 2: fission creates different sets of starting conditions to the regenerating fragments and thus making them ‘siblings’. (C) Case 3: the naïve fragment is a descendent of the experienced head fragment which still retains the memory. While similar to case 2, the head fragment in this case remains the ‘parent’ of the tail fragment and does not undergo any major process of resetting of past experiences. (D) Case 4: the organisms that result from fission are truly identical if neuronally-encoded memories are shared between the clones (‘myself’).

The provocative idea, which demands additional study, that certain memories in planaria survive decapitation, presents a useful opportunity for debate. We present a few hypothetical scenarios, not mutually exclusive, that will allow us to ask whether upon fission a planarian that is derived from the head fragment can consider the regenerated fragment that arises from its cut-off tail fragment as ‘my twin’, ‘my sibling’, ‘my child’ or ‘myself’ (Fig. 1).

Case 1: If upon fission and regeneration the two resulting fragments are identical in every aspect (genetically, epigenetically), and if experiential brain-encoded memory is erased (Rilling, 1996), then the two individuals can be considered clones or truly ‘identical twins’.

Case 2: If asymmetric fission non-homogenously establishes epigenetic differences, including in the process of brain development, so that the two planarians have different starting conditions to life, then the two individuals are ‘siblings’, not identical twins.

Case 3: If a memory is specifically acquired in the brain, and if upon beheading the worm that regenerated the tail retains the memory, while the worm which regenerated a new brain does not, then perhaps the birth of the new naïve tissue (e.g. a new brain) is the birth of a new generation. The naïve fragment is the ‘child’ in this case, and the experienced fragment is the ‘parent’.

Case 4: If some neuronally acquired memories can still be maintained in a new worm regenerated from the tail piece of the original worm (Corning, 1966; Shomrat and Levin, 2013), then the underlying mechanisms for transgenerational transmission of memories, if found, could produce two individuals that are true clones in every way, similarly to the situation in Case 1. Moreover, since in this hypothetical scenario the two fragments share their neuronally produced memories, one fragment could consider the other fragment as ‘myself’.

## In which cases does the term ‘generations’ apply?

Diverse phyla of animals regenerate body portions after damage (Birnbaum and Sánchez Alvarado, 2008). In the phylum Cnidaria, this capability is the rule rather than the exception, with some of its members possessing remarkable regenerative capabilities. For instance, isolated medusa muscle cells can undergo transdifferentiation and regenerate an entire organism (Schmid and Alder, 1984). This attribute of cnidarians had already been documented more than two centuries ago by Abraham Trembley, who first described the regeneration of *Hydra*, a capability which had hitherto been supposed unique to plants and fungi (Galliot, 2012). In some cnidarians this regenerative ability has parallels to regeneration in planaria; for example, in *Hydra* the somatic stem cells that drive regeneration express piRNAs, similarly to planarian neoblasts (Juliano et al., 2013).

In general, even traditional reproduction can be considered an ultimate form of regeneration, where an entire organism is re-created from a single cell of the adult (the egg). In this section we wish to expand the discussion, and consider whether the questions that were raised above in regard to planaria apply to other organisms as well. We have discussed how memory could be transferred between individuals as a result of the blending of boundaries between development and inheritance in asymmetrically dividing animals. Since diverse organisms use different mechanisms to procreate and to store information, it is worthwhile to reflect on the broader definition of the terms ‘generations’ and ‘memory’, and the interaction between these processes.

## ‘Generations’ of dividing cells

When cells are grown in culture in the laboratory, the ‘generation time’ of the culture is frequently tracked and different ‘generations’ display different phenotypes, which often accumulate in later ‘generations’ (Merrill, 1998; Niida et al., 1998). In addition to amassing damage (e.g. shortening of telomeres, mutations), when cells divide, whether in a multicellular organism or in unicellular organisms, certain memories can be inherited through mitosis; daughter cells can stably maintain the memory of different cellular activities initiated in the parental cell when the cytoplasm is split in two, through different feedback mechanisms (Campos et al., 2014; Wang et al., 2013). The ability to maintain expression patterns of the parental cells in the daughter cells is a key to development and differentiation (Hobert, 2011). Not all the information is preserved; for example, DNA replication and the ensuing dilution of the histones present a challenge for preservation of chromatin marks (which epigenetic marks are maintained after S phase is still an open question in the field) (Budhavarapu et al., 2013; Lanzuolo et al., 2011; Probst et al., 2009). Histone variants are being removed off replicating DNA, and the new histones are being deposited on the newly synthesized DNA as the replication fork progresses. Which molecules or information enable, in cases when this type of memory is indeed preserved (Gaydos et al., 2014), to decorate the histones of the daughter strands with the same post-translational modifications that were present on the histones of the template DNA? This is a very active field of investigation and there are currently no definitive answers (Campos et al., 2014). In contrast, re-establishment of DNA methylation patterns on the newly synthesized DNA is fairly well understood (the process depends on the maintenance activity of the DNA methyltransferase, DNMT1) (Kar et al., 2012). Despite the mechanistic ambiguity, it is clear that certain environmental changes can elicit responses that are memorized over cell division; maintenance of acquired properties in a bacterial and yeast population, such as fast responses to different environmental conditions or nutrients, was shown to persist over long periods of time (and thus through generations) (Lambert and Kussell, 2014).

## Generations in plants

Plants provide a striking example of evolution of organisms which lack a designated population of stem cells that will become germ cells. One of the aspects of plant cell biology that distinguishes between regeneration in plants and planaria is the ability of certain plant cells to dedifferentiate or transdifferentiate in response to environmental cues. There is no single source of cells for new tissues in plants, as apart from meristems (structures consisting of pluripotent stem cells) there are various undifferentiated cell populations in the plant that can propagate and differentiate (Aichinger et al., 2012). Additionally, certain somatic cells may transdifferentiate to grow various plant tissues (Sugimoto et al., 2011). The ability of plant cells to dedifferentiate is under tight regulation of cell-specific gene expression, in the absence of which flower meristems and even embryos may develop spontaneously from somatic tissues (Bowman et al., 1992; Horst et al., 2016; Ikeuchi et al., 2015).

The same processes that regulate the dedifferentiation of somatic cells are part of normal plant growth and development. For instance, the presence of an apical meristem inhibits the development of axillary meristems (Leyser, 2003). The absence of a nearby meristem, either caused by its removal or by the growth of the plant, will reduce this inhibition and allow the development of dormant meristems, or the development of undifferentiated cells into meristems or the formation of new meristems from dedifferentiated somatic tissue (Leyser, 2003).

**Fig. 2.**
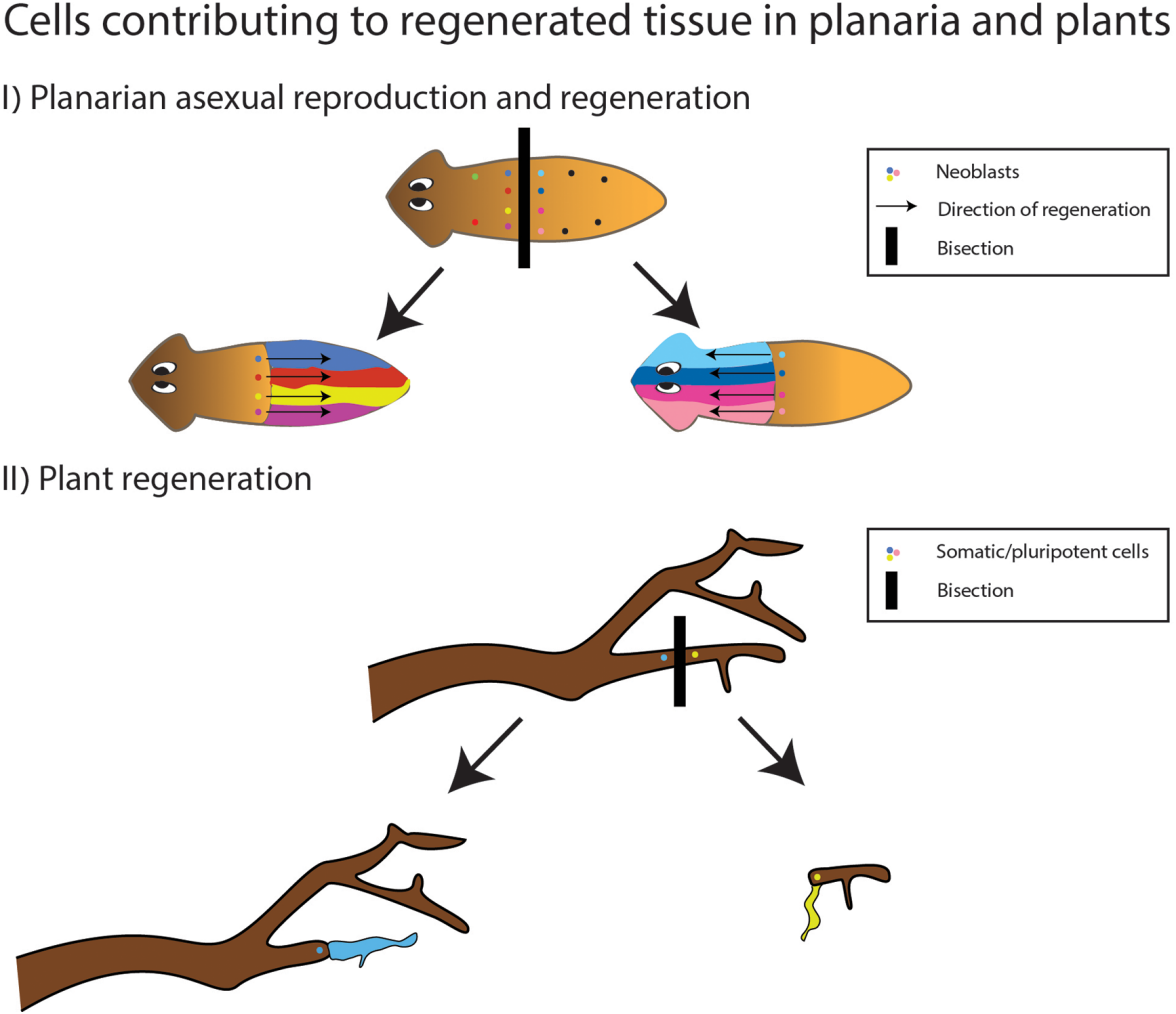
**The effects of cellular mosaicism on regenerated tissues in planaria and plants.** (I) After fission or bisection, each neoblast in the formed blastema may differ in its genetic and epigenetic content, and contribute to the variation in the regenerated tissue, resulting in difference between and within the regenerated fragments. (II) After a break in the plant tissue, various somatic cells may regenerate plant tissue. The newly grown tissue may differ genetically and epigenetically due to environmental effects on its originating somatic cells.

Whether through a natural or artificial process, cloning can result from injury or detachment of a portion of the plant. However, formally, the definition of ‘generations’ in plants refers to the completion of a ‘life-cycle’, from embryo to adult (Bai et al., 2000; Harada et al., 2001). Since in vegetative reproduction there is no passing through an embryonic stage, does the individual which grows out of the severed part constitutes a new generation? In addition to vegetative reproduction, some plant species reproduce through the formation of plantlets on somatic tissues (Kulka, 2006). This process is defined as asexual reproduction, due to the formation of an embryo. Although the ‘progeny’ is a clone, and there is no germline involved, the embryo can mark a border between generations, due to its position in the plant's life cycle (Bai, 2015). Also in the case of clonal reproduction in plants, clones may differ depending on the fragmented tissue from which it was grown. This type of variation is termed somaclonal variation, and may be caused by genetic or epigenetic differences in the cells from which the clone develops (Wang et al., 2013). The notion that mosaicism can give rise to differences between regenerated parts is schematically described in Fig. 2.

Though it is also possible to clone plants without the mediation of an embryo using its regenerative properties, and despite the fact that this action yields two individuals, this form of cloning is not commonly referred to as asexual reproduction. Plants display a full arsenal of epigenetic mechanisms, including the ones described in relation to planaria, such as histone modifications, DNA methylation, and small RNA-induced RNAi (Dunoyer et al., 2010; Habu et al., 2001; Kaeppler et al., 2000). Moreover, plants have the ability to amplify heritable small RNAs that are used for gene silencing using RNA-dependent RNA polymerases (similarly to *C. elegans* nematodes) (Rechavi et al., 2011) and small RNAs can also direct DNA methylation in the nucleus. These mechanisms enable preservation of transgenerational epigenetic memory, in addition to maintenance of epigenetic memory after cell division (Castel and Martienssen, 2013). Additionally, as dedicated structures such as plasmodesmata connect different plant cells, diffusible epigenetic markers in somatic cells may affect the stem cells that regenerate, produce embryos or germ cells. It is possible that the mechanisms, which may create variability in planarian clones, could contribute to somaclonal variation. Indeed, in addition to prevalent genetic mosaicism (Gill et al., 1995), some ‘epimutations’ that originate in plant ancestors can become stable over hundreds of generations (Ong-Abdullah et al., 2015).

Since different reproduction processes in plants, as described above, do not require passage through an embryonic step (that defines which individual is the ‘parent’ and which is the ‘child’), the relationship between the two resulting individuals is somewhat ambiguous, and bears many similarities to the relationship between two regenerated planarian fragments.

## Generations in sexually reproducing animals

In sexually reproducing animals, the ‘clear’ conceptual classification of individuals along a lineage to distinct generations is allowed due to the discrete steps of meiosis and fertilization. In mice and humans, extensive erasure of epigenetic information that originated in the parent by germline and embryo ‘reprograming’ takes place (Hajkova, 2011). Reprogramming of DNA methylations, for example, has been shown to be critical for totipotency (Messerschmidt et al., 2014; Surani, 2001). Since reprogramming entails the erasure of ancestral ‘memories’, it might be suggested that reprogramming could serve to define ‘time zero’, when the separation of the new generation from the parent takes place. However, in sexually reproducing animals, for example *C. elegans*, it is not clear to what degree epigenetic marks undergo ‘reprograming’ (Anava et al., 2014); *C. elegans* do not methylate cytosines, however some ancestral small RNAs and chromatin modification were explicitly shown to persist in the progeny, for multiple generations (Gaydos et al., 2014; Rechavi et al., 2011, 2014).

It is not yet known which type of memories/reactions can persist across generations in sexually reproducing animals (not even in organisms where this is an intensely studied question, such as *C. elegans*). Thus, it is not clear in what sense animal pedigrees could be considered to form an epigenetic ‘continuum’ which stretches over time, and to what extent each member in a lineage is a ‘true epigenetic individual’.

It is probable that the degree of ‘epigenetic continuity’ between generations of different animals differ, since different animals appear to diverge in the mechanisms that are at their disposal for maintaining epigenetic memory across generations. For example, no mammals are currently known to share the ability of *C. elegans* to amplify heritable small RNAs using RNA-dependent small RNAs (Rechavi et al., 2011). The notion of a clear-cut generation is an abstract concept, however, in sexually reproducing animals a new generation can be identified solely based on meiosis and fertilization – the definition should not be based on epigenetic resetting.

## Suggested experiments

We proposed that asymmetric fission might encourage variation between the individuals that regenerate from the fragments. Here we detail experiments that could add support to this hypothesis.

### Maintenance of epigenetic markers derived from fragment tissue

Each fragment has a gene expression pattern that is specific to its morphology; however, when it is removed from an intact worm and forced to regenerate new structures, it must remodel these gene-regulatory events on top of new anatomy (i.e. a trunk fragment containing largely intestine must generate new positional information to specify head and tail regions). The incomplete reprogramming of these markers may lead to their maintenance throughout the complete animal (Thomas and Schötz, 2011). In other words, an organism that regenerates from a tail may be more ‘tail-like’ than one that regenerates from a head. This can be assessed after a single fission event by comparing the gene expression of the resulting whole organisms and those of the specific tissue of the fragment. While the continued success of regeneration over millions of fission events through the history of planaria suggests that such history or enrichment cannot accumulate indefinitely, it is possible that some limited amount of ‘recent’ history of spatial origin is kept. It will be especially interesting to identify persistent molecular or biophysical markers of anatomical (positional) history (Carlson, 1983; Chang et al., 2002) in fragments that originate in different regions of one-headed versus permanently two-headed worms, to decipher the algorithm by which blastema cells of any fragment type decide which structures to generate at each wound surface.

### Maintenance of bioelectric gradients derived from fragment tissue

The main open questions concern what changes (transcriptional, chromatin-level, or bioelectrical) distinguish a trunk fragment from a wild-type worm (destined to make one head) and an anatomically-normal trunk fragment from a two-headed worm (which will make two heads). Examination of bioelectric state (using fluorescent reporters of voltage distributions) (Adams and Levin, 2012; Oviedo et al., 2008), transcriptional profiling, and chromatin state analysis must be used to understand what is different about these fragments. Quantitative models must be developed to explain how stable states can be stored, and edited, in physiological circuits (Cervera et al., 2015; Law and Levin, 2015; Levin, 2014a).

### Maintenance of behavioral memories across regenerative reproduction

To determine how and where memory may be stored outside the brain during head regeneration, it would be necessary to first optimize training protocols (Abbott and Wong, 2008; Blackiston et al., 2010; Inoue et al., 2015; Nicolas et al., 2008; Pagán et al., 2012), capitalizing on more ecologically-salient stimuli and learning paradigms to achieve high-throughput induction of robust learning. The key experiments would be to assess the persistence of memory in fragments of different sizes, anatomical locations and body compositions. A variety of molecular and biophysical tools now exist to establish suppression screens targeting various pathways, to begin to probe the mechanisms necessary for imprinting of the memory upon a newly-regenerating brain (Aoki et al., 2009; Gentile et al., 2011; Sheı˘man and Kreshchenko, 2015).

## Conclusions

In planaria, and other organisms that reproduce by fission, producing and maintaining variation between fragments after asymmetric division may be adaptive (much like the beneficial increase in variation following sexual reproduction and recombination). Therefore, the theoretical ability of asymmetric division to create variability in an otherwise isogenic population could be considered as a tool for producing evolutionary progress. Thus, asymmetric fission is a mechanism that challenges our current view of what defines the temporal axis of evolution, since epigenetic processes, environmental cues, biochemical gradients and generation of a complete individual from a community of cells can generate natural variation, without requiring so called ‘distinct’ generations. It is likely that we have only begun to glimpse the prevalence and variety of long-term memory in somatic tissues during lifespan and across reproduction throughout phyla. The continued future analysis of such instructive interactions is likely to have profound implications for understanding evolution. Moreover, a mature understanding of these fascinating processes will drive numerous applications in regenerative medicine and bioengineering that exploit the rich informational plasticity of tissues for the rational control of form and function.
